# There are only four basic modes of cell death, although there are many ad-hoc variants adapted to different situations

**DOI:** 10.1186/s13578-018-0206-6

**Published:** 2018-02-01

**Authors:** Xingde Liu, Wenxiu Yang, Zhizhong Guan, Wenfeng Yu, Bin Fan, Ningzhi Xu, D. Joshua Liao

**Affiliations:** 1 Department of Cardiology, Guizhou Medical University Hospital, Guiyang, 550004 Guizhou People’s Republic of China; 2 Department of Pathology, Guizhou Medical University Hospital, Guiyang, 550004 Guizhou People’s Republic of China; 3 Key Lab of Endemic and Ethnic Diseases of the Ministry of Education of China in Guizhou Medical University, Guiyang, 550004 People’s Republic of China; 4 Laboratory of Cell and Molecular Biology & State Key Laboratory of Molecular Oncology, National Cancer Center/Cancer Hospital, Chinese Academy of Medical Sciences and Peking Union Medical College, Beijing, People’s Republic of China

**Keywords:** Apoptosis, Necrosis, Programmed cell death, Stress induced cell death, Senescence, Regeneration, Scar, Inflammation

## Abstract

There have been enough cell death modes delineated in the biomedical literature to befuddle all cell death researchers. Mulling over cell death from the viewpoints of the host tissue or organ and of the host animal, we construe that there should be only two physiological cell death modes, i.e. apoptosis and senescent death (SD), as well as two pathological modes, i.e. necrosis and stress-induced cell death (SICD). Other death modes described in the literature are ad-hoc variants or coalescences of some of these four basic ones in different physiological or pathological situations. SD, SICD and necrosis kill useful cells and will thus trigger regeneration, wound healing and probably also scar formation. SICD and necrosis will likely instigate inflammation as well. Apoptosis occurs as a mechanism to purge no-longer useful cells from a tissue via phagocytosis by cells with phagocytic ability that are collectively tagged by us as scavengers, including macrophages; therefore apoptosis is not followed by regeneration and inflammation. The answer for the question of “who dies” clearly differentiates apoptosis from SD, SICD and necrosis, despite other similarities and disparities among the four demise modes. Apoptosis cannot occur in cell lines in vitro, because cell lines are immortalized by reprogramming the death program of the parental cells, because in culture there lack scavengers and complex communications among different cell types, and because culture condition is a stress to the cells. Several issues of cell death that remain enigmatic to us are also described for peers to deliberate and debate.

## Background

There have been many cell death modes described in the biomedical literature [1–3], far more than enough to confuse probably all the experts who dedicate their careers to the research of cell death mechanisms [4]. Those who are not fully dedicating themselves to cell death research must be even more befuddled. Readers of this essay are encouraged to test themselves and ask around whether they or any of their acquaintances are familiar with all of the 34 cell death modes listed below that are collected by us from the literature: accidental cell death [2], regulated cell death [2], senescence, necrosis, regulated necrosis [5, 6], type I cell death [2, 7, 8], type II cell death [2, 7, 8], type III cell death [2, 7, 8], cannibalistic cell death [9], programmed necrosis [10], aponecrosis [11], netosis [12], necroptosis [12, 13], apoptosis, mitochondrial apoptosis [14], intrinsic apoptosis [15], extrinsic apoptosis [15], non-apoptotic cell death [6], non-apoptotic regulated cell death [16], mitotic catastrophe [17, 18], degeneration [19, 20], parthanatos [21], entosis [9, 22], cornification [23], methuosis [24], oncosis [25], paraptosis [26], anoikis [27], pyroptosis [28], ferroptosis [29], phagoptosis [30], caspase-independent apoptosis [31, 32], cell death independent of caspases [33], and excitotoxicity [34, 35]. Besides these 34 demise modes, there must be many other less frequently used terms not listed, of which we are unaware. There are also many other types of cell death that do not belong to any of the abovementioned modes and have not been extensively studied. For instance, in a heartbeat cells can be burnt to death or killed by strong acids or bases, and chewing a grape also kills the grape’s cells instantly in the mouth. Moreover, some cellular activities, including micropinocytosis, macropinocytosis [36], phagocytosis, autophagy, etc., are often considered as cell death mechanisms, although they per se are not cell death but can lead to death of the cells if the activities are severe enough and persistent. As a good example, autophagy is developed to recycle (and thus save) resources via a cellular cannibalism, i.e. consumption of a cell’s own components, for the cell to survive such a situation wherein nutrients are scarce, and, when the cell is cancerous and fast-growing, to build new cells more economically. However, overconsumption of cellular components will lead to cell death.

Although there seem to be many different ways for cells to die, if we step back and look at cell death more distantly, from the viewpoints of the host tissue or organ or the host animal, we may not be so baffled. Even just looking at the cell per se will be less perplexing than a close-up look at the molecular details. After a long meditation on the relationship between the cell that is about to die and its host tissue/organ or its host animal, which most studies do not emphasize, we extrapolate that there are only four basic cell death modes, including two physiological ones, i.e. senescent death (SD) that is death from cellular aging, and apoptosis, as well as two pathological ones, i.e. necrosis and stress-induced cell death (SICD) [37]. Of course, this classification has culled away those caused by extreme physical or chemical factors, such as the aforementioned “burnt-to-death” one, on which studies may not have much clinical value (although studies of damage by milder physical and chemical factors do have value). Other than these four, the remaining demise modes listed above do exist, but they are ad-hoc variants or amalgams of some of these four basic ones in different situations. The appearance of so many ad-hoc death mechanisms is largely because cells in a creature of evolutionarily high level on one hand have allegiance to the host tissue, or the creature that wants these cells to die for its ultimate best interest, but on the other hand the cells are smart and selfish and try their best to survive all different stressed situations. Restated, the interaction between these two conflicting or paradoxical facets of animal cells leads to the large variety of ad-hoc survival mechanisms and death mechanisms of cells. In this essay we expound our musings on these aspects to challenge several basic notions that have been ingrained in, and have formed the mainstay of, cell death research. Modes of cell replication that are so often associated with cell death are briefly introduced as well.

### Modes of cell proliferation in physiological situations

In a creature high on the evolutionary “tree of life”, one important feature is that each cell type has a physiological total number. Another trait is that in most organs and tissues, cells come and go by replication and death whereas, in some other cell types such as neurons and cardiac myocytes, cells are no longer capable of replication after the animal reaches a certain age. There are two types of cell replication, i.e. direct proliferation and compensatory proliferation [38–41]. In humans, direct proliferation occurs during body growth before and during puberty or occurs in some special situations, such as in obesity that requires more adipose cells to store extra fat, or in pregnancy during which cell proliferation occurs in the uterine and mammary tissues. Compensatory proliferation, commonly referred to as “regeneration”, occurs when some cells have reached the end of their lifespans, i.e. when cells die from aging, which is defined herein as SD, and thus the host tissue or organ needs new cells to make up the physiological number of cells. Some people estimate that in a human body 60 billion cells die every day [42], although some others estimate that one million cells die every second [43–46], which is 86.4 billion cells per day. Of course, some of these many deaths may be due to a pathological reason such as SICD that will be described later. Therefore, the human body needs to yield 60–86.4 billion new cells per day to compensate for the cell loss.

### Modes of cell death in physiological situations

In an animal of evolutionarily high level, physiologically there are only two major modes of cell death, i.e. apoptosis and SD. The term “apoptosis” was given by Kerr et al. in 1972 [47], but this type of cell death was described over one and half centuries ago [8, 48], although the descriptions by different research-ancestors were not quite the same. In evolutionarily high animals, many cells are no longer needed during or after certain developmental or physiological states, such as during digit individualization in the human embryo, post-pubertal involution of the thymus, postpartum involution of the uterus, post-lactating (post-weaning) involution of mammary glands, etc., as some of us have reviewed before [37, 49]. Apoptosis is the mechanism used by the animal to get rid of these cells that are no-longer useful to it and thus are redundant. The animal requests these obsolete cells to die via a pre-determined procedure, or a “program”, thus making this death mode regarded as a “programmed cell death”.

One hallmark of apoptosis is that the cell death is physiological and should not cause any harm to the host tissue and certainly not to the entire organism. In animals, this is achieved by mobilizing macrophages or other cells that have phagocytic ability, collectively coined as “scavenger cells” [49, 50], to engulf the dying or dead cell before its decomposition to dregs that contaminate the host tissue environment and release immunogenic materials to incite inflammation. This phagocytosis occurs actively and requires communication between scavengers and the cell which is to die, dubbed as “apoptoting cell” by some of us [49], via such as “find-me” and “eat-me” signals [50] and via other signals from the apoptoting cell to promote the scavengers’ survival [51] and migration [52]. This phagocytosis of apoptoting cells as an iconic feature of apoptosis was first described by Kerr et al. [47] as well as by Schweichel and Merker [7], but recently it seems to have been studied again in more detail under the umbrella of “entosis” [9]. Sending out “find-me” and ‘eat-me” signals from apoptoting cells to scavengers indicates that apoptosis is a suicidal event. Moreover, these traits also mean that apoptosis has fully evolved only in those animals equipped not only with macrophages that have a huge capacity for phagocytosis but also with blood and lymphatic circulation systems that allow macrophages to migrate from distant sites to the suicidal cells. However, considering apoptosis “evolutionarily developed” also acknowledges that simpler apoptosis mechanisms should already have existed in those animals lower on the life tree. Indeed, caenorhabditis elegans has no blood nor lymphatic circulation system but already has an apoptosis mechanism, since the dying or already-dead cells can be engulfed by their neighboring cells [53]. Because carcinogenesis can be regarded as an atavistic process, a tumor can be regarded as an evolutionarily-lower organism that parasitizes the host patient [37, 54, 55]. Therefore cancer cells in a tumor lump live “physiologically” in the host body and may have an apoptosis mechanism simpler than that in the patient’ cells. Nevertheless, complex communication and coordination between a predator and its prey implies that a fully developed apoptosis is a highly programmed event, which is initiated by a motivation of the animal’s body to eliminate outmoded cells and is terminated at the complete clearance of the cell corpse inside the scavenger, with preservation of the host tissue environment as the basic pre-condition [37, 49, 50, 55]. For example, after pups wean, lactating mammary epithelial cells no longer have value to the dam and thus need to be purged from the breast, but this massive cell death should not be detrimental to the dam, i.e. should not cause inflammation and scar formation in the breast. Any mode of cell demise sans this motivation and this corpse clearance but with inimicality to the host tissue is not an authentic and fully-developed apoptosis, which, unfortunately, is rarely emphasized as one of the icons of apoptosis in relevant publications. Actually, as has been repeatedly wrangled before by some of us [37, 49, 50, 55], pure apoptosis occurring in such as aforementioned in vivo models has received little attention because most studies avowed to be on apoptosis are virtually on SICD [37] that will be described later.

**Fig. 1:**
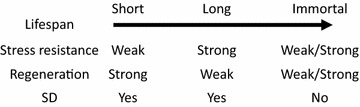
Hypothetical reciprocal relationship between the lifespan on one hand and the abilities to regenerate and to resist stress on the other hand. The abilities of immortalized cells (such as cancer cells or cell lines) to regenerate or resist vary greatly, depending on the cell types, but they should not undergo SD

In an animal such as in the human, all normal cells, without exception, undergo a three-phased aging procedure, i.e. (1) proliferation for a certain number of cycles, (2) loss of the replication ability and then quiescence for a certain period of time, (3) and then dying and death. This means that all normal animal cells have lifespans, although the lifespans of different cell types are different. For instance, the lifespans for presumptive naive T cells (phenotype CD45RA+) and memory T cells (CD45RO+) in the human are calculated to be 3.5 years and 22 weeks, respectively [56]. Some cell types in the human, such as neurons and cardiac myocytes, lose their proliferation ability during childhood but then have the longest lifespans, basically living until the person dies. This usually means that these cells have a strong ability to resist various stress insults during the decades of the person’s life. Partly due to this advantage, i.e. this resistance, those cells that have lost replication ability do not develop tumors (tumors originated from heart muscles and neurons were initiated during the embryonic stages and are manifested as childhood diseases) [40, 41, 54, 55, 57]. On the other hand, some other cell types such as keratinocytes in the skin and mucosal cells in the gastro-intestinal tract have relatively short lifespans, i.e. die quickly, but meanwhile they also have a strong ability for regeneration to make up for the quick cell loss, collectively manifested as a high rate of cell turnover [54, 58, 59]. Actually, in many organisms including bacteria, slowly-proliferating cells survive better than their fast-proliferating counterparts [54, 60]. This hypothetical reciprocal relationship between the lifespan, on the one hand, and the ability to regenerate and to resist stress on the other hand, as summarized in Fig. 1, deserves more thorough research.

Cellular aging is often referred to as senescence, the meaning of which, however, is often ambiguous in the literature, as many peers define it as “irreversible growth arrest” [61], which does not necessarily mean that the cell will die. To avoid equivocality, we refer to cell death from aging as SD. Since in apoptosis it is those no-longer useful cells that die, those cell types that have lost regeneration ability but have long lifespans may not die from apoptosis because they are, in fact, useful, or, more correctly, indispensable. Instead, since the host creature wants these very useful cells to live and function as long as possible, they can only die from SD in a physiological situation, besides the pathological death that will be narrated later. Cells that die of SD will also be gulped down by scavenger cells, just like apoptotic cells, and thus should not be inimical to the host tissue. Of course, those apoptotic or senescent cells that reside on a body surface (such as in the skin) or near a cavity (such as in the intestinal tract) will directly fall out and thus will not be scavenged. These cells will not be discussed herein, as a caveat already has been given before [50].

### Absence of apoptosis in cell culture

As some of us have repeatedly addressed before [37, 49, 50, 55], for several reasons an authentic apoptosis as above defined never occurs in cell lines cultured in Petri dishes. First, cell lines are all immortalized, established by reprogramming the death program of the parental cells [57], whereas study of apoptosis is for the purpose of determining the original, unchanged death program. Actually, in our opinion, all immortal cells, including all tumor cells, do not have a death program because they do not have a lifespan, according to the definition of “immortality”, and thus cannot die from a programmed procedure with suicide as its essence. Even if, as described in so many publications, immortal cells still have a death program established by reprogramming the normal cells’ death program, studies using these cells with an already-changed death program can only provide us with already-altered mechanisms. More complicatedly, different cell lines, no matter whether they are derived from spontaneous tumors or are manmade in the lab using such as viruses, are immortalized via different mechanisms, as summarized before by one of us [57]. Therefore, different cell lines will have differently-reprogrammed death programs and will likely provide us with different mechanisms or pathways of cell death. This is one of the reasons why so many demise mechanisms have been identified, and likely many more are waiting for us to identify them, although in our opinion most of these mechanisms are actually ad hoc variants of SICD that will be described later. Second, a genuine apoptosis is initiated for a twofold purpose, i.e. clearance of useless and thus redundant cells under a condition of preserving the host tissue in an intact status. However, cell lines are autonomous and thus no longer allegiant to the host tissue and animal and, in a culture dish, have no reason to care about whether their environment is polluted or not by their cellular shreds. In short, cell lines in Petri dishes have no motivation to keep their environment undisturbed. Third, most cell culture systems used for apoptosis studies involve only one single cell line in the Petri dish, thus lacking scavenger cells and in turn lacking complex communications among different cell types. These missing communications include those between apoptoting cells and scavengers via such as “find-me” and “eat-me” signals, between apoptoting cells and their healthy sibling cells and even cells in distant organs to “discuss” which and how many cells are really redundant and need to be eliminated, and between the non-apoptoting cells and scavengers via such as “don’t eat me” signals to protect useful siblings from being mistakenly predated by scavengers [50]. Fourth, apoptosis consists of two parallel procedures, one occurring in the suicidal cell and the other in the scavenger [50]. The mid and later parts of apoptosis occur inside the scavenger and involve its enzymes to dispose of the prey. These two procedures, each in a different cell but in parallel with the other, are highly coordinated via the aforementioned cell–cell communications even before the suicidal cell is wolfed down by the scavenger. In those studies with only one single cell line as the only player in the culture dish, one procedure is lacking, making impossible the coordination between the two. Because of these reasons and some others that have been mentioned before [50], even if a programmed cell death occurs in vitro as described in so many publications, it occurs in an unusual situation, study of which can only provide us with unusual mechanisms and pathways that do not actually occur in an animal’s body [50]. At least, in our body there normally is no any single immortal and autonomous cell, and thus the mechanisms identified in cell lines have no relevance to normal persons, while we should keep it in mind that apoptosis is evolutionarily developed, and thus is a mechanism for the normal.

### Modes of cell proliferation in pathological situations

In pathological situations, animal cells may proliferate without a need for compensation for cell loss, resulting in hyperplasia, which is a pathological phraseology for the existence of extra (redundant) cells, usually with enlargement of the affected organ as a sequel. For example, some chemicals such as phenobarbital [62, 63] or lead nitrate [64] can induce proliferation of hepatocytes, resulting in liver enlargement [41]. Aberrant expression or mutation of some genes, usually oncogenes, may also coerce the affected cells to replicate directly [55]. Direct proliferation occurring in these situations is a pathological event and, as it results in an excess of cells, apoptosis ensues as a recovery procedure to remove the redundant cells [40, 41]. For this reason, many transgenic mice expressing a proliferation-driving oncogene show high proliferation rates but low carcinogenic efficiencies, because many mutation-bearing cells have been swept out via apoptosis soon after they were produced [38, 40, 41, 65]. It goes without saying that benign or malignant tumor cells can also proliferate continuously, as tumor in pathology textbooks is defined as “uncontrolled cell growth”, or “autonomy in replication”.

### Modes of cell death in pathological situations

**Fig. 2:**
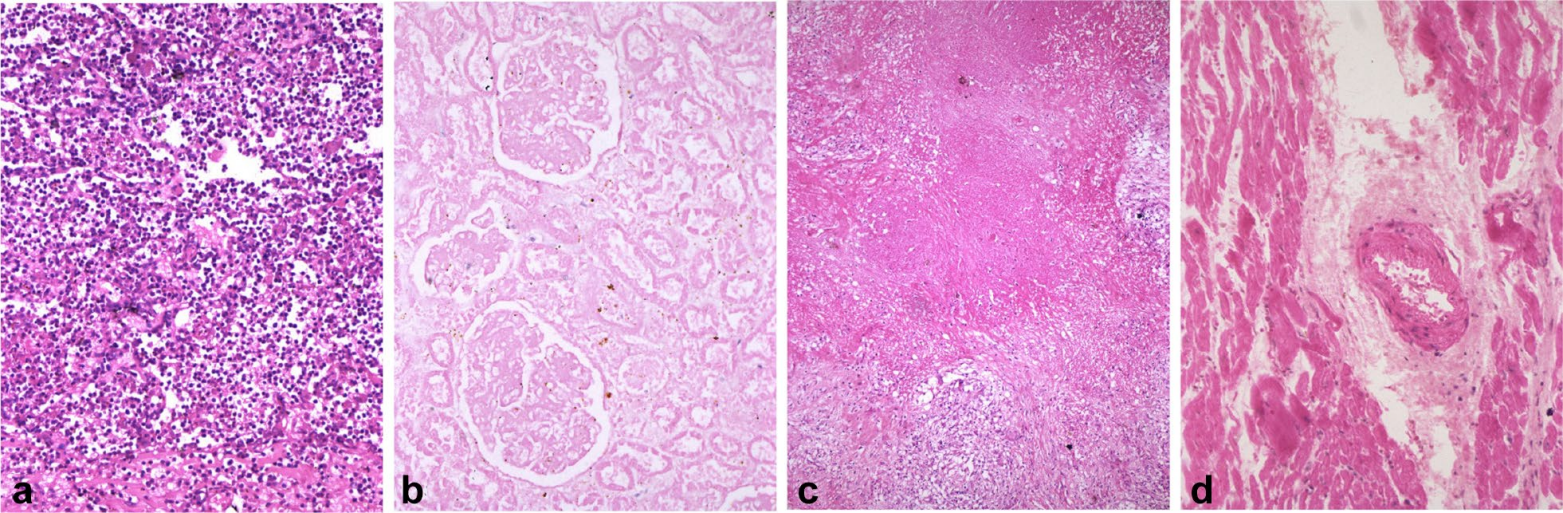
Several common necrosis types in human tissues. **a** An area of liquefactive or lytic necrosis from a lung abscess, which is fraught with inflammatory cells. **b** An area of coagulative necrosis from a kidney infarction showing that all necrotic cells have lost their outlines and cellular morphology but the renal histology still remains intact. Note that there are few inflammatory cells infiltrating into this large necrotic area. **c** An area of caseation necrosis from a tuberculous lymph node showing the lack of massive infiltration of inflammatory cells. **d** An area of fibrinoid necrosis from a heart with rheumatic disease showing the lack of extensive infiltration of inflammatory cells

Severe stress can kill cells instantly, and it usually is an exogenous stress, such as a bacterial infection or an infarction in which blood supply to part of an organ is blocked. Cell death resulting from a harsh stress usually abounds, occurs within a short spell, and does not require participation of scavenger cells, unlike apoptosis and SD. However, when cell corpses have decayed to smithereens and then release immunogenic components, scavenger cells will be mobilized to engulf the cellular dregs as part of the inflammation. This type of cell death is called necrosis, which has been well delineated in pathology textbooks for centuries. Necrosis as a pathological event commences with many irreversible changes in the nucleus, mitochondria and other organelles of the affected cells. After these cellular changes, the necrotic cells may merge, making the outlines of individual cells indistinct and together often forming a focus of coarsely granular, amorphous, or hyaline material. Common types of necrotic lesions that need to be taught to every medical student worldwide are shown in Fig. 2.

Sometimes, a stress, especially when derived from the cell per se, may not be taxing enough to cause immediate cell death but may be capable of turning on some intrinsic suicidal mechanism of the cell. This mode of cell demise is referred to as SICD [37]. For example, during physiological cell turnover, i.e. when cells are regenerating to compensate for those that have died of aging, i.e. SD, DNA mutation may occur as a contingency in some proliferating cells. The cell will first arrest its proliferation, usually at the G1 or S phase of the cell cycle, to repair the DNA. However, sometimes the mutation is irreparable; in this case, the cell usually turns on a demise program to commit suicide, so that the mutation will not be passed to progeny cells to be hereditary. It goes without saying that chromosomal fragmentation [66–68] or other forms of mitotic catastrophe [17, 18], with much greater genomic damage than single mutations, are extreme examples of this type of SICD that are usually discerned in cancer cells. Sometimes blood cells as frontier fighters against micropathogens like bacteria and viruses are infected by bacteria or viruses but cannot eliminate them. In this case the cells will turn on a death program to commit suicide, so that they will not carry and thus spread the pathogens to other body sites [54]. This “kill your foe or kill yourself” phenomenon, which is often seen in movies, occurs daily in our body. Similar to such micropathogen infections, there are many other types of exogenous stress that are severe but still insufficient to kill cells instantly and thus trigger an endogenous stress to initiate SICD. This is typically discerned in patients receiving radiation or some chemotherapeutic agents that cause substantial DNA damage not only in cancer cells but also in some normal cells; the damaged DNA in turn serves as an endogenous stress to precipitate SICD. In a nutshell, many cells in those animals higher on the life tree have an allegiance to the animal’s body and will die via SICD, if such sacrifice is needed to maintain the life of the animal in a stressed situation, which is a common trait, acquired evolutionarily, of many organisms, especially in the animal kingdom [37, 54, 55].

When stress induces death of only a small number of cells, scavenger cells are able to purge them all from the tissue or organ. This situation resembles apoptosis and thus is dubbed as “stress-induced apoptosis-like cell death (SIaLCD)” [37]. Actually, it may be possible that the number of necrotic cells is small as well, since sometimes harsh stress may affect only a few cells and not necessarily kill many. Moreover, the necrotic cells may be scavenged before they decompose to cellular dregs, making necrosis indistinguishable from SIaLCD. However, necrosis is still a homicide, since the cells are killed, but do not die from an intrinsic suicidal program. In addition, this engulfment may be an action of macrophages alone and may not involve signals such as “find-me” and “eat-me” from the dying cells, thus differing from that in apoptosis. The fact that all multicellular animals have an intention to minimize the deleterious effect of dead cells on their bodies makes macrophages dual-functional in inflammation: on one hand they are one of the important inflammatory components. On the other hand they function to prevent inflammation from occurring by swiftly gobbling up dying or dead cells to prevent putrefaction of cell corpses to cellular shards. When cells that die of SICD are superabundant and inundate scavenger cells, many cell corpses will decompose and release various immunogenic cellular materials to agitate inflammation, first locally and then systemically. This subtype of SICD is coined as “stress induced necrosis-like cell death (SInLCD)” [37].

Because in necrosis and SICD it is those useful cells that die, the unaffected normal cells will regenerate to compensate for the cell loss and to heal the wound. In both necrosis and SInLCD, sometimes the cell death is massive and persistent and goes beyond the regeneration capacity, in such as chronic hepatitis B virus infection wherein the viruses not only constantly kill hepatocytes but also impede the regeneration of the still-alive hepatocytes. In this situation, cells of connective tissue, mainly fibroblasts, will step into help the wound-healing by forming a scar, which in the liver infected by the hepatitis B virus manifests as cirrhosis. Generally speaking, regeneration and wound healing follow SCIaLCD, SCInLCD and necrosis but scar formation may only follow SInLCD and necrosis, due to an immense loss of useful cells [37].

### The actual meaning of the well-characterized caspase-cytochrome c pathway

The above-described SICD has been well-characterized but, haplessly, it is misconstrued as apoptosis in most relevant studies. Indeed, the caspase-cytochrome c pathway of cell death has been widely accepted as a typical apoptosis pathway. However, several issues of this pathway have been neglected: at its physiological location (the inner membrane of the mitochondrion), cytochrome c (Cyt-c) participates in ATP production to power the cell, thus sustaining the cell’s life and making it actually an oncoprotein. Only when mitochondrial permeability is changed, causing leakage of Cyt-c from the inner membrane to the cytoplasm, which is a purely pathological (stressed) event and is likely induced by a form of stress (such as a chemo drug), does it cause cell death and only then does it function as a tumor suppressive protein [69]. In other word, the caspase-Cyt-c pathway not only is caused by a stress but also requires relocation of Cyt-c from its normal cellular location to an abnormal one wherein, and only wherein, it can bind to other death-driving proteins to cause cell death [69]. Thus, it occurs only in a pathological situation. Little, if any, evidence has been shown that (1) Cyt-c can cause death without a form of stress involved, such as a culture condition wherein usually only 10% serum is available, (2) Cyt-c leaking-out from the mitochondrial inner membrane can also be a physiological event, and (3) at its physiological location (within the inner membrane of mitochondrion) Cyt-c can cause cell death as well. Actually, other than Cyt-c, there are many proteins that are compartmentalized in a sort of organelle of the cell because they function differently in physiological and pathological situations; lysosomal enzymes are another example, as the enzymes will digest out the cell and kill it once they have leaked out from the lysosome to the cytoplasm. Therefore, the well-characterized caspase-Cyt-c pathway of cell death is actually a mechanism of SICD, but not of apoptosis, in our opinion [69]. However, much of the literature is correct about that it is indeed a “mechanism of cancer remedies” that, in our opinion, is SICD and not apoptosis, either. Of course, it remains possible that apoptosis lends its mechanism to SICD to deal with various pathological situations, and we just don’t know it, since studies on true apoptosis are very insufficient with such aforementioned in vivo models as post-weaning involution of mammary glands and postpartum involution of the uterus [50]. In other words, it is currently unclear whether SICD borrows the death program from apoptosis or uses one different from that of apoptosis.

**Fig. 3:**
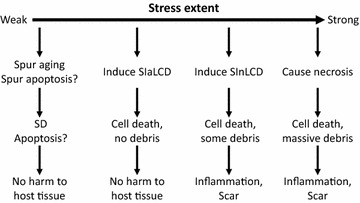
Relationships between different extents of stress and different cell death modes. Very mild stress may impel cellular aging, leading to an earlier SD of the affected cells. Stress may also hasten apoptosis, although this conjecture still lacks concrete supporting evidence since few in vivo studies focus on unadulterated apoptosis. A stronger stress may cause SIaLCD and an even stronger stress may cause SInLCD with more dead cells exceeding the clearance capacity of scavenger cells and decaying to cellular shards to agitate inflammation. A severe stress will directly kill cells via necrosis

We are all familiar with a phenomenon that a stressful life ages people more quickly. Therefore, stress, even when it is too mild to directly kill cells via necrosis or SICD, may be an impetus for cellular aging leading to SD, probably in part by affecting telomere length [70]. We opine, with trepidation, that with the extent of stress increasing, the same form of stress to the same type of cell will first prod SD, and then SIaLCD, followed by SInLCD and necrosis, as depicted in Fig. 3.

### Similarities and disparities among apoptosis, necrosis, SICD and SD

**Fig. 4:**
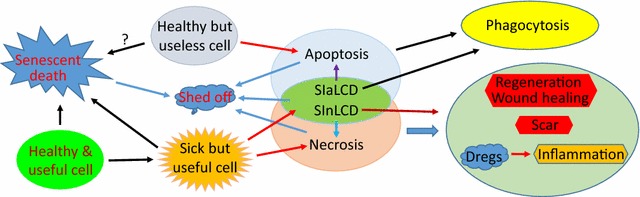
Illustration of the relationships among apoptosis, SD, SICD and necrosis. It is the healthy but useless cells that undergo apoptosis, whereas it is the useful but damaged cells that die of SICD (either sIaLCD or SInLCD) or necrosis. Useful cells, either healthy or damaged ones, can age and eventually die of SD, but whether obsolete cells also undergo SD is an intriguing question that remains murky, because these cells may be removed much more efficiently via apoptosis. Apoptotic and SIaLCD cells will be phagocytosed whereas SInLCD and necrotic cells will decompose to cellular dregs that cause inflammation. Moreover, SInLCD resembles necrosis that will cause regeneration and wound healing, probably in association with scar formation, but these activities do not follow SIalCD and apoptosis

Apoptosis as a suicide, and necrosis as a homicide, are irreconcilable to each other. SICD as another death mode resides between apoptosis and necrosis with many similarities and disparities to the two, as summarized in Fig. 4 and Table 1, often causing confusion or being mistaken as apoptosis or necrosis. First, SICD occurs to the useful cells and is a pathological event, which resembles necrosis but starkly contrasts with apoptosis. Second, because in SICD it is the useful cells that die, cell regeneration, wound healing and probably also scar formation ensue, which again resembles necrosis but contrasts with apoptosis that eliminates archaic cells and therefore does not trigger regeneration. Because of the need for regeneration and wound healing, SICD involves complex communications between the dooming cells and the surrounding healthy cells on such important issues as how many cells need to be regenerated, when and where the minted cells should emerge, as well as whether fibroblasts need to step into help heal the wound. Although necrosis is also followed by regeneration, its homicidal nature and the resulting swiftness of cell death may not allow for such complicated cell–cell communication. Since SICD is a programmed suicidal procedure, it resembles apoptosis by enticing scavenger cells to dispose of the cell corpse via complicated communications between the predator and the prey to coordinate the time and the location of the predation. Both apoptosis and SICD may involve communications between the dying cells and their healthy siblings, but this aspect has gained little attention and few explorations. Third, if in SICD the death tally is exceedingly high and goes beyond the clearance capacity of scavengers, i.e. in a situation of SInLCD, inflammation succeeds as aforementioned, which resembles necrosis but differs from apoptosis. Fourth, apoptosis can only occur in vivo but SICD and necrosis can occur in cell culture as well. Actually, most mechanisms and pathways described in the literature for “apoptosis” have involved stress of every kind, cell lines of every type and cell culture systems of every sort, and therefore are actually for SICD, as aforementioned. Reiterated more clearly, SICD is well studied with much of the mechanism(s) well illustrated from cell lines in culture while unadulterated apoptosis is poorly studied with the mechanism(s) largely unknown.

**Table 1:** Similarities and disparities among apoptosis, SD, SICD and necrosis

Death mode	Who dies?	Who kills?	Prog.	Nature	Normal or not	Inflam.	R & H	Scar	Scavenged	Cell line	Cell–cell communication		
											w Scav.	w Nor.	S vs N
Apoptosis	Normal but useless cell	Animal body	Yes	Suicide	Physiological	No	No	No	Evident	No	Yes	Yes	Yes
SD	Normal but useful cell	Worn-out	Yes	Suicide	Physiological	No	Yes	No	Evident	No	Yes	Yes	No
SICD													
SIaLCD	Useful but damaged cell	Exo/endo stress	Yes	Suicide	Pathological	No	Yes	No	Evident	Yes	Yes	Yes	Yes
SInLCD						Yes		Maybe					
Necrosis	Normal and useful cell	Exo stress	No	Homicide	Pathological	Yes	Yes	Maybe	Not evident	Yes	No	No	No

Primary cells in culture dish may undergo SD but cell lines may not as they are immortalized

*Exo/endo* exogenous or endogenous, *Prog.* programmed, *Inflam.* inflammation, *R & H* regeneration and would healing, *w Scav.* with scavenger cells, *w Nor.* with normal sibling cells, *S vs N* scavengers with normal sibling cells

SD is a suicide of useful cells, which resembles SICD but differs from apoptosis. Because of the neat coordination in the living body, the tally of death from SD should not be so high as to glut the scavengers’ capacity. Therefore, usually SD is not associated with inflammation, which resembles apoptosis and SIaLCD but differs from SInLCD and necrosis. For those cell types that retain a regeneration ability, regeneration follows SD as it is the useful cells that die, making SD similar to SICD and necrosis but dissimilar to apoptosis. Since, as aforementioned, apoptosis, as well as regeneration following SD, SICD and necrosis, require different spectra of cell–cell communication and interaction, SD has similarities and differences with apoptosis, SICD and necrosis in this aspect.

### Many cell death modes and survival pathways as ad-hoc variants

In our opinion, of the many cell death modes described in the literature, some are ad-hoc variants of apoptosis or SD in different physiological situations, while most others are ad-hoc variants of SICD in different pathological situations or in different cell lines because SICD resides between apoptosis and necrosis. For instance, cornification is apoptosis occurring in skin [23], whereas SICD is a better idiom to summarize such death modes as “regulated necrosis”, “necroptosis”, etc., that manifest both necrotic and apoptotic features. Cells often die via SICD, because they always try to utilize all possible means to survive a particular stress although they still die eventually because their death is due to the organism’s iron will to deal with the particular stress or because they cannot defy the stress. Owing to this property of “using all available mechanisms to survive a particular situation”, cells survive initially and then die differently among different particular situations, creating many ad-hoc survival pathways and in the meantime leaving us with many ad-hoc modes of cell death. For example, pyroptosis is SICD of macrophages in which pyrogens can be released to cause hyperthermia [28]. The parlances like “caspase-independent apoptosis” and “cell death independent of caspases” may be superfluous, since we surmise that authentic apoptosis in an animal may indeed not involve caspases originating from the dying cell itself, because macrophages as professional cell disposers have professional enzymes, including caspases, to dispose of their prey [50]. Although few studies have been conducted to explore the mechanisms of authentic apoptosis in vivo, there is some in vivo evidence supporting this conjecture: post-weaning involution of mouse mammary glands does not show aberrant activation of caspases and their downstream effector protein PARP-1 [71], and still occurs normally in caspase-3 knockout mice [72]. Moreover, apoptotic death of mammary tumor cells in c-myc transgenic mice is actually associated with a decreased expression of Cyt-c [73]. However, a caveat needs to be given that these many ad-hoc variants of the four basic cell death modes are still meaningful and worth exploring as they reflect cell death, mainly SICD, at different particular circumstances, understanding of which is an important scientific footing for precision medicine or personalized medicine.

Stress can directly kill cells (necrosis), can turn on intrinsic death program of cells (SICD), and can goad aging-caused cell death (SD), depending on the extent of stress and the cell type, as different cell types can withstand different extents of stress. For instance, as an adverse event, a given radiotherapy or chemotherapy can directly kill some normal cells (necrosis) but can only cause SICD or spur SD of some other normal cells while having no effect at all on a third set of normal cells, creating heterogeneity of cell death in a given tissue or organ. Actually, heterogeneity of cell death is a common phenomenon when a tissue encounters a strong stressor [74]. It is also possible that a given stress causes death of the same cell via combined mechanisms, including SD, SICD and necrosis. In our cogitation, it is not that necrosis can also be a programmed event but it is because SICD is misconstrued as necrosis. Also, it is not that apoptosis may be immunogenic as well, as alleged in many studies [2], but it is because SICD is misconstrued as apoptosis.

### Remaining conundrums

All animals, including humans, have been programmed in their nuclear and probably also mitochondrial genomes to die eventually, and all cells in an animal will die along with the animal itself, if not earlier. There hitherto has not been any way to immortalize an animal, and not even an organ, but individual cells can be easily reprogrammed to be immortal, either spontaneously as bespoken by benign or malignant tumor cells appearing in humans, or intentionally as cancer researchers often do in labs. Therefore, the program of cellular SD is not the program of aging of the organ or the animal. A related question that is still under debate is whether prokaryotic and unicellular eukaryotic cells undergo aging, since these unicellular organisms, typically bacteria, maintain their species by constant cell division [58, 75]. Cancer cells are immortal and, even after the patient has died, can survive perpetually as cell lines, in which situation individual cancer cells resemble such unicellular organisms as bacteria that keep dividing to maintain themselves. What still awaits clarification is whether cancer cells and even benign tumor cells also age, and thus also undergo SD, since in so many studies senescence is another nomenclature of cellular aging and since there are plentiful publications describing senescence of cancer cells [76]. Reporting SD of tumor cells, spontaneously or induced by an implementation, seems to oppose their immortal nature and thus seems preposterous, because it says that “immortal ones will still age and die of aging”. Alternatively, one can strictly define “senescence” as “irreversible growth arrest” and disconnect it from cellular aging and cell death. In our logic, stress of any kind, such as an irradiation or a chemotherapy, is unable to induce or accelerate SD of immortal cells, such as cancer cells and various cell lines, either in vivo or in vitro, although it can kill these cells via necrosis or SICD. Restated, a remedy causes only SICD or necrosis, but not SD, of cancer cells that are immortal.

We are also contemplating over whether apoptosis, SD and SICD are really programmed events as stated in this and almost all other relevant articles. A program is a pre-determined procedure, which in our opinion opposes the fact that most cells in animals are very plastic and can easily adapt to different changes in their microenvironment with a purpose for survival or for a better life, just like those of us who crave for a better life and increased longevity. The fact that there have been so many ad-hoc modes of programmed cell death identified demonstrates the extreme flexibility of demise programs. If a program can be changed easily, i.e. can adapt easily to every slight change in the microenvironment, it is actually not a program that is pre-determined.

Another question over which we have for long been pondering is whether apoptosis as a pure physiological event developed evolutionarily is encoded by a cellular structure, irreversible change of which is responsible for the irreversibility of the cell death procedure. Or is apoptosis just like aging and type 2 diabetes that, unlike most other biological functions, lack a structural basis as some of us have wrangled before [58]? This is because no such cellular structure has been identified yet that is uniquely responsible for authentic apoptosis.

What bedevils us the most is such a notion that necrotic cells putrefy to release immunogenic cellular materials to instigate inflammation. Although this is true in lytic necrosis and probably at a late stage of some other types of necrosis, Fig. 2 shows a very striking phenomenon in which there rarely are immune cells in large areas of common necrosis types, even when all necrotic cells have already lost their outlines and have fused together. This trait seems dissonant with the above description that inflammation is one of the consequences and hallmarks of necrosis, but few articles discuss this incongruity.

## Concluding remarks

In this essay we propose that the fraternity of cell death research should clarify the use of “apoptosis” to the animal kingdom by emphasizing it as a particular cell demise mechanism used by animals to remove older cells without causing damaging reactions, mainly inflammation and scar formation, to the host tissue or organ. “Involution type of cell deaths”, such as post-weaning involution of lactating mammary glands, are typical examples of unadulterated apoptosis. We opine that there only two basic physiological cell death mechanisms, i.e. apoptosis and SD, and only two basic pathological cell death modes, i.e. SICD and necrosis. SICD dwells between apoptosis and necrosis with similarities and differences between the two, which often makes it misconstrued as apoptosis or necrosis. SICD can be split to SIaLCD and SInLCD, depending on whether or not the dead cells can be swiftly engulfed by scavengers. More complicatedly, SICD can be easily adapted to different particular situations to become different variants that are named differently in the literature. Authentic apoptosis does not occur in cell lines whose original death program has been reprogrammed to make the cells immortal, and does not occur in cell culture that is a stress to cells, uses cell lines, and lacks other cell types as other important players of apoptosis. Many similarities and disparities among apoptosis, SD, necrosis and SICD delineated in this essay should help peers to distinguish these four basic cell death modes, and the variants derived from them, from one another. Particularly, apoptosis has evolutionarily developed to purge no-longer useful cells from the host tissue or organ, which is a yardstick to differentiate itself from SD, SICD and necrosis that cause death of useful cells and thus are followed by regeneration, wound healing and probably also scar formation. Some notions, which have been ingrained in cell death research and firmly entrenched in the mind of many peers but may be preposterous, are also described in this essay as unanswered conundrums for future exploration and for peers to debate.

## Abbreviations

Cyt-c: cytochrome c
SD: senescent death
SICD: stress-induced cell death
SIaLCD: stress-induced apoptosis-like cell death
SInLCD: stress induced necrosis-like cell death
